# Construction and validation of a risk prediction model for early acute kidney injury in asphyxiated neonates

**DOI:** 10.3389/fped.2026.1666855

**Published:** 2026-04-29

**Authors:** Huiyue Zhang, Jinju Shi, Xianghui Huang, Tianyi Zhou, Hongzhu Cai, Yanni Xu, Shaoru Zheng, Cuimin Su

**Affiliations:** 1 Jinjiang Municipal Hospital (Shanghai Sixth People's Hospital Fujian), Jinjiang, China; 2 Fujian Key Laboratory of Neonatal Diseases, Xiamen, China; 3 Children’s Hospital of Fudan University (Xiamen Branch), Xiamen Children’s Hospital, Xiamen, China; 4 Department of Maternal and Child Health, School of Public Health, Peking University, Beijing, China

**Keywords:** asphyxiated neonates, acute kidney injury (AKI), risk prediction model, near-infrared spectroscopy (NIRS), renal injury biomarkers

## Abstract

**Objective:**

This study aims to construct a prediction model based on the Naive Bayes classifier to predict the risk of acute kidney injury (AKI) in asphyxiated neonates in the early stage.

**Methods:**

The subjects were 79 asphyxiated neonates born and treated at Hospital from September 2022 to December 2024. By collecting clinical data, measuring renal tissue oxygen saturation (RrSO_2_), and detecting relevant biomarkers, and by combining SMOTE oversampling technique and Recursive Feature Elimination Cross-validation (RFECV) algorithm, six key variables (Cystatin-C, RrSO_2_, oliguria, lactate, β_2_-microglobulin, and creatinine) were selected to build the prediction model. Interpretable machine learning methods were used to explain the established model, and a nomogram for calculating the disease probability of the research subjects was plotted.

**Results:**

The model achieved an accuracy of 0.929, sensitivity of 0.889, and specificity of 1.000 on the training set; on the testing set, it achieved an accuracy of 0.826, sensitivity of 0.846, and specificity of 0.800.

**Conclusions:**

The study shows that this model can provide a reliable reference for the early assessment of AKI risk in clinical practice, thus helping improve the prognosis of asphyxiated neonates. Future research needs to expand the sample size and conduct multicenter validation to further optimize the model.

## 1 Introduction

Perinatal asphyxia is a serious and prevalent health problem affecting newborns globally. It has been reported that in developed countries, there are 1–2 cases of asphyxia in every 1,000 live births. The incidence rate is much higher in low- and middle-income countries, ranging from 5 to 15 cases per 1,000 live births (1). As a common critical emergency during the perinatal period, neonatal asphyxia refers to a pathological reaction in which, during childbirth (2), due to various etiologies, the newborn fails to establish normal respiration, thereby triggering hypoxia, acidosis, and tissue metabolic dysfunction.

When neonates suffer from asphyxia, the body initiates the diving reflex to redistribute blood, prioritizing the blood supply to vital organs such as the heart, brain, and adrenal glands. This causes the blood flow to the kidneys to decrease rapidly, making them highly susceptible to renal injury. Relevant studies have shown that neonatal asphyxia can lead to dysfunction in multiple organs of the neonate, with the incidence rate of heart, brain, and kidney dysfunction being as high as about 70% (3). Due to the immaturity of the neonatal kidneys and their poor tolerance to hypoxia, acute kidney injury (AKI) is likely to occur after asphyxia. AKI refers to a series of clinical syndromes caused by a rapid decline in renal function within a short period of time, mainly manifested as a decrease in the glomerular filtration rate (GFR) (4). The incidence and mortality rates of AKI in the neonatal period are relatively high. Past studies have shown that in neonates with multiple organ dysfunction caused by perinatal asphyxia, the incidence of AKI is approximately 35%–55% (5).

Early detection and treatment of renal damage are crucial for improving the prognosis of asphyxiated neonates, as AKI is an independent risk factor for mortality and morbidity (6). However, early AKI lacks specific clinical manifestations, making it difficult to detect in a timely manner in clinical practice, thus delaying treatment opportunities and potentially progressing to acute renal failure, which increases the mortality rate of the infant (7). Therefore, assessing the risk of AKI as early and accurately as possible and taking effective intervention measures in a timely manner are of vital significance for reducing the incidence of AKI and improving the prognosis of asphyxiated neonates.

Currently, there is a lack of simple and effective methods for predicting the risk of early AKI in clinical practice. Traditional diagnostic criteria mainly include clinical symptoms such as oliguria and hematuria in neonates, as well as the elevation of serum creatinine (SCr) and blood urea nitrogen (BUN) levels. However, these indicators often show atypical signs in the early stages of neonatal renal injury, and SCr and BUN can only reflect overall renal function damage, failing to accurately determine early renal injury. By the time changes in SCr and BUN are observed, renal function damage has usually reached a certain level, missing the optimal treatment window.

Near-infrared spectroscopy (NIRS) can monitor renal tissue oxygen saturation (RrSO_2_) in real-time to reflect the oxygen metabolism status of renal tissue; serum Cystatin—C, lactate, urinary α1-microglobulin (α1-MG), β_2_-microglobulin (β_2_-MG), and other biomarkers are closely related to renal function and have potential application value in the early diagnosis of renal injury. The Bayesian model, as a powerful data analysis tool, can integrate multiple factors and has unique advantages in dealing with uncertainty and small sample data.

Based on the above background, this study aims to collect clinical data from asphyxiated neonates, measure RrSO_2_ using NIRS, and detect relevant biomarkers to construct a risk prediction model for early AKI in asphyxiated neonates using the Naive Bayes classifier. It is expected to provide a reliable reference for the early and accurate assessment of AKI risk in clinical practice, thereby enabling timely detection of AKI. The findings of this study will help improve neonatal fluid management and drug selection, leading to better prognosis.

## 2 Materials and methods

### 2.1 Clinical data

Neonates with asphyxia who were born and treated at Hospital from September 2022 to December 2024 were selected as the subjects of this study.

According to the Canadian 2009 neonatal asphyxia diagnostic criteria (8), severe asphyxia was Apgar score at 1 min of 0–3; heart rate of 100 beats per min at birth and decreasing or steady; respiration absent or gasping; color poor; muscle tone absent. Mild or moderate was Apgar score at 1 min of 4–7; normal respiration not established within 1 min but heart rate of 100 beats per min; some muscle tone present; some response to stimulation. Based on the these criteria, the neonates were divided into a mild asphyxia group with 57 cases and a severe asphyxia group with 22 cases. There were 43 males and 36 females, with a gestational age of (36.95 ± 2.93) weeks and a body weight of (2.732 ± 0.73) kg. Among them, 34 neonates with asphyxia had AKI, and 45 neonates with asphyxia did not have AKI.

Inclusion criteria: (1) Compliance with the diagnostic criteria for neonatal asphyxia. (2) Complete clinical data and acceptance of relevant examinations. (3) Approval by the Ethics Committee of Hospital, with informed consent signed by the parents or guardians of the asphyxiated neonates, and the researchers' commitment to protect the content of the investigation and personal privacy of the subjects.

Exclusion criteria: (1) Congenital renal dysplasia. (2) Genetic metabolic diseases. (3) Renal dysfunction caused by drugs, infections, jaundice, etc.

### 2.2 Methods

#### 2.2.1 Data collection

Basic information of the neonates (gender, gestational age, birth weight, degree of asphyxia, umbilical cord abnormalities, placental abnormalities), and maternal conditions, including age, mode of delivery, pregnancy complications (gestational diabetes, gestational hypertension), intrauterine distress, and amniotic fluid contamination.

#### 2.2.2 Renal tissue oxygen saturation detection method

NIRS measurement: The non-invasive RrSO_2_ detection was performed within 6 h after birth using a near-infrared tissue oximetry monitor (model: EGOS-600B). The probe was placed 2–3 cm to the side of the spine on the neonate's waist, avoiding bones and large blood vessels, based on anatomical landmarks and renal ultrasound positioning. Continuous measurement was conducted for 10 min, and the average value was taken as the RrSO_2_ value.

#### 2.2.3 Urine collection and detection method

Urine was collected from asphyxiated neonates within 24 h after birth, and oliguria was defined as urine output less than 1 mL/kg/h. Fresh urine (10 mL) was collected using a urine collection bag and centrifuged at 1,500 r/min for 10 min. The HITACHI 7600 Automatic Analyzer was used to measure urinary β2-MG and α1-MG levels by immunoturbidimetry. The detection process was strictly carried out according to the standard operating procedures and the instructions of the reagent kit.

#### 2.2.4 AKI diagnosis and grouping

The AKI diagnosis criteria proposed by the Kidney Disease Improving Global Outcomes (KDIGO) (9) were used: (1) An increase in serum creatinine of more than 26.5 µmol/L within 48 h from the baseline at birth. (2) An increase in serum creatinine to more than 1.5 times the baseline within 7 days. (3) Urine output < 0.5 mL/(kg·h) for more than 6 h. All neonates were divided into AKI and non-AKI groups based on whether AKI occurred.

### 2.3 Statistical methods

Children were divided into two groups based on whether they had AKI. For continuous data (gestational age, birth weight, maternal age, laboratory indicators), *t*-tests were used; for categorical data (gender, degree of asphyxia, oliguria, umbilical cord abnormalities, mode of delivery, intrauterine distress, amniotic fluid contamination, etc.), chi-square tests were employed.

Given the class imbalance in the study population, with a higher proportion of children in the AKI group, SMOTE (Synthetic Minority Over-sampling Technique) oversampling was utilized to correct the imbalance. SMOTE is a classic algorithm for addressing class imbalance in datasets, which synthesizes new minority class samples based on the feature space distribution and similarity of existing minority class samples (10), thereby balancing the dataset and enhancing the model's ability to recognize and classify minority class samples. In this study, SMOTE oversampling was applied to balance the proportion of children in the two groups, ensuring the model's accurate identification of features from different classes.

During the variable selection phase, to precisely identify the best variable combination for AKI prediction, the Recursive Feature Elimination Cross-validation (RFECV) algorithm was employed. RFECV is an effective feature selection method that uses random forests to assess and rank variable importance (11). It recursively eliminates the least important variables from the full set, generating subsets of different sizes. Cross-validation is performed on each subset, and the subset yielding the best model performance is selected, achieving automatic feature selection. In this study, the final variable combination was chosen based on the principle of maximum accuracy. That is, in each iteration of the RFECV algorithm, the model's predictive accuracy under the current variable combination was calculated and used as the evaluation criterion. Ultimately, the variable combination that maximized model accuracy was selected for subsequent modeling.

In the modeling phase, the variables selected by the RFECV algorithm were used to model whether children had AKI, employing a Naive Bayes classifier. Specifically, the entire study population was divided into training and testing sets in a 7:3 ratio. SMOTE resampling was applied to the training set, and the RFECV algorithm was combined with the Naive Bayes classifier to model the data using five-fold cross-validation. The model's predictive performance was then validated on the original, unresampled testing set, with ROC curves drawn and predictive efficacy calculated.

Subsequently, this study employed interpretable machine learning methods to conduct in-depth analysis of the established model, exploring the variable importance and the specific contribution of each variable to the model's prediction results. Finally, to further enhance the clinical application value of the model—specifically, to calculate the probability of AKI based on patient conditions—this study developed a nomogram based on the established model, aiming to boost its application value.

## 3 Results

### 3.1 Basic characteristics of the study subjects

The descriptive statistical analysis of the baseline characteristics of the 79 subjects in this study. The results show that in terms of gender composition, males accounted for 43%. The average gestational age of the children was 36.95 weeks, and the average birth weight was 2.73 kg. The cesarean section rate was 33%, and the average length of hospital stay for the children was 13.09 days. Compared with the Non-AKI group, the AKI group had a higher proportion of mothers and children with Severe Asphyxia, Umbilical Cord Abnormality, Placental Abnormality, and Fetal Distress, and the differences were statistically significant. However, the incidence of gestational diabetes was higher in the Non-AKI group (Table 1).

**Table 1:** Basic characteristics of the study subjects.

Variable	Overall	AKI	Non-AKI	*P*
	Mean (SD)	Mean (SD)	Mean (SD)	
Total (%)	79	34 (43.04)	45 (56.96)	
Male (%)	43 (54.4)	19 (55.9)	24 (53.3)	1.000
Gestational week	36.95 (2.93)	37.02 (2.77)	36.90 (3.08)	0.859
Birth weight	2.73 (0.73)	2.83 (0.72)	2.66 (0.74)	0.323
Cesarean section (%)	33 (41.8)	18 (52.9)	15 (33.3)	0.129
Hospitalization days	13.09 (13.01)	15.29 (16.33)	11.42 (9.66)	0.192
Severe asphyxia (%)	22 (27.8)	16 (47.1)	6 (13.3)	0.002
Umbilical cord abnormality (%)	30 (38.0)	18 (52.9)	12 (26.7)	0.032
Placental abnormality (%)	17 (21.5)	12 (35.3)	5 (11.1)	0.021
Fetal distress (%)	30 (38.0)	18 (52.9)	12 (26.7)	0.032
Gestational diabetes mellitus (%)	14 (17.7)	3 (8.8)	11 (24.4)	0.133
Gestational hypertension (%)	5 (6.3)	1 (2.9)	4 (8.9)	0.543
Cystatin—C	1.80 (0.55)	2.10 (0.56)	1.57 (0.42)	<0.001
RrSO_2_	47.14 (8.03)	42.09 (5.82)	50.96 (7.38)	<0.001
Oliguria (%)	31 (39.2)	27 (79.4)	4 (8.9)	<0.001
Lactic acid	5.21 (3.47)	7.02 (4.28)	3.83 (1.76)	<0.001
β2-MG	5.38 (5.30)	8.07 (5.66)	3.35 (3.99)	<0.001
Creatinine	65.42 (14.34)	64.47 (11.83)	66.13 (16.07)	0.612

### 3.2 Results of variable selection and importance ranking by RFECV

**Figure 1:**
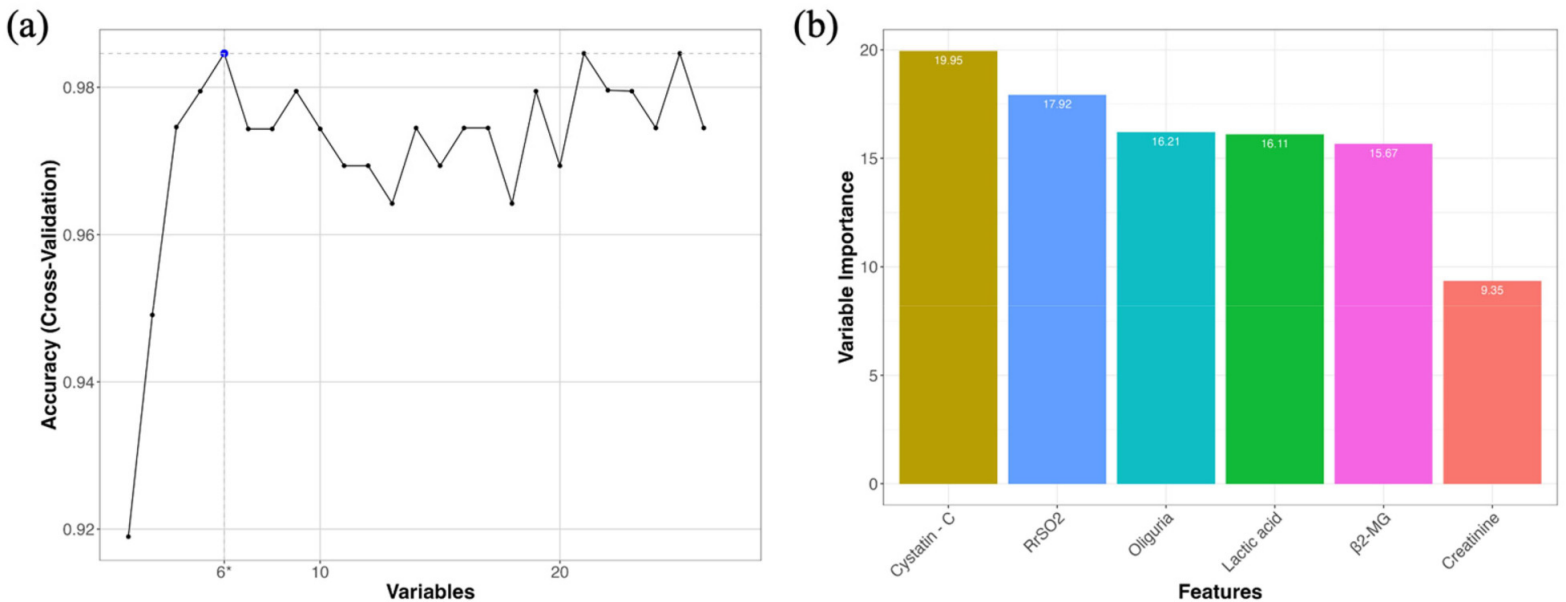
Predictive accuracy of RFECV under different combinations of variables and results of variable selection. **(a)** Variation in cross-validated model accuracy with the increasing number of candidate variables. The highest predictive accuracy was observed when six variables were included, indicating the optimal feature subset. **(b)** Relative importance of the six variables selected by RFECV, including Cystatin C, rSO_2_, oliguria, lactic acid, β2-MG, and creatinine.

The results of the best variable combination selection for the model using the RFECV algorithm. The results indicate that when the number of model variables is low, the model's predictive accuracy gradually increases with the addition of variables. The accuracy peaks at 0.9846 when the model contains six variables. Beyond this point, further increases in the number of variables lead to a slight decrease in predictive accuracy. When the number of variables exceeds 13, the model's predictive accuracy fluctuates. Therefore, the six variables identified when the model size is six are selected for subsequent modeling, as they achieve the highest predictive accuracy with the fewest variables (Figure 1a). The results of the best variable combination and their importance rankings selected by RFECV. The six variables selected are Cystatin-C, RrSO_2_, Oliguria, Lactic acid, β_2_-MG, and Creatinine. Among these, Cystatin-C has the highest importance score of 19.95, while Creatinine has the lowest importance score of 9.35 (Figure 1b).

### 3.3 Prediction performance of the AKI prediction model established by five-fold cross-validation

The prediction performance of the AKI prediction model, established using the variables selected by RFECV and combined with a Naive Bayes classifier through five-fold cross-validation, on the original training and testing sets without resampling. When applied to the training set, the model achieved an accuracy of 0.929 (95% CI: 0.827, 0.980), with a sensitivity of 0.889, specificity of 1.000, positive predictive value of 1.000, and negative predictive value of 0.833. When applied to the testing set, the model's accuracy was 0.826 (95% CI: 0.612, 0.951), with a sensitivity of 0.846, specificity of 0.800, positive predictive value of 0.846, and negative predictive value of 0.800 (Table 2).

**Table 2:** Confusion matrix of the established AKI prediction model in the training and test sets.

Dataset	Prediction	Reference		Total
		AKI	Non—AKI	
Training set	AKI	20	4	24
	Non—AKI	0	32	32
	Total	20	36	56
Testing set	AKI	8	2	10
	Non—AKI	2	11	13
	Total	10	13	23

**Figure 2:**
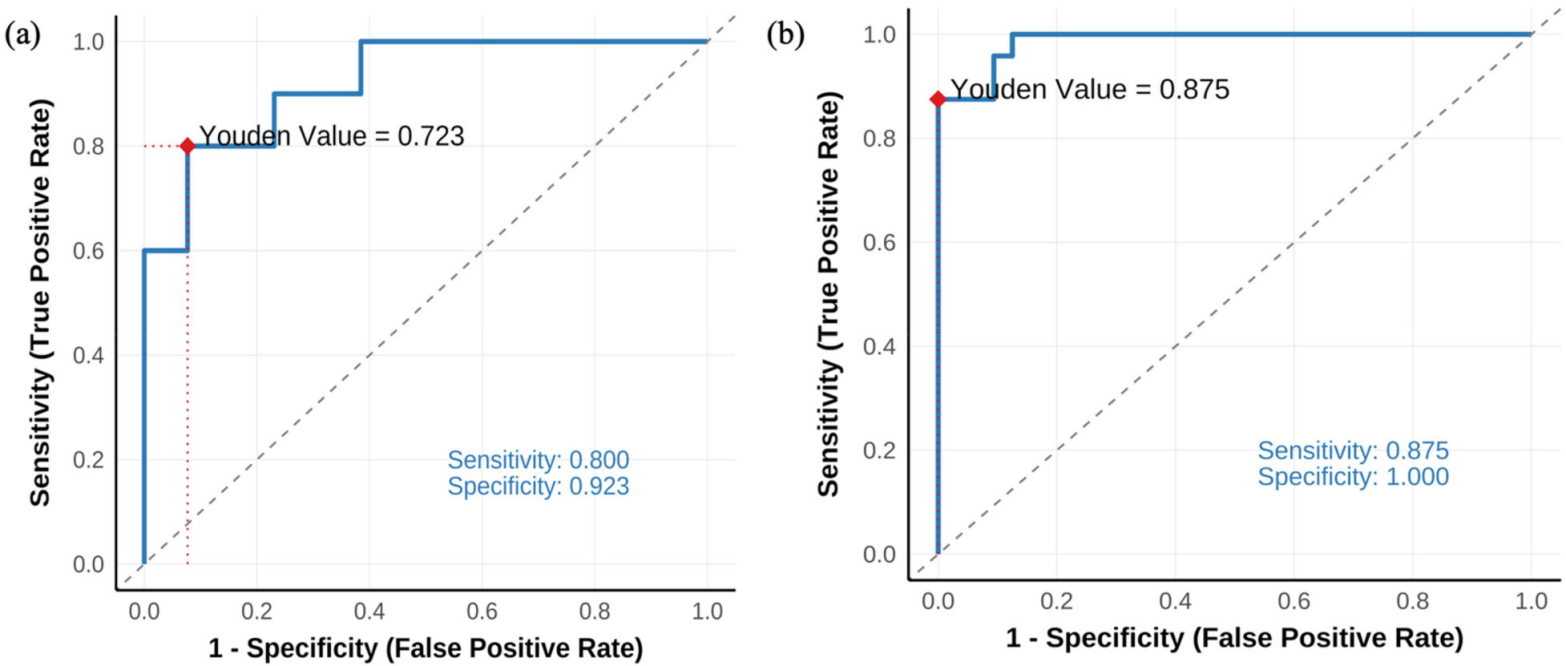
ROC curves of the established AKI prediction model in the original training and test sets. **(a)** In the test set, the optimal cutoff identified by the Youden index yielded a sensitivity of 0.800 and a specificity of 0.923. **(b)** In the training set, the optimal cutoff yielded a sensitivity of 0.875 and a specificity of 1.000. The diagonal dashed line indicates random classification performance.

The ROC curves of the AKI prediction model on the original training and testing sets without resampling. Figure 2a shows the results on the training set, where the model's predictive performance is better, with an AUC of 0.987. Figure 2b shows the results on the testing set, where the model's predictive performance is slightly lower than that on the training set, with an AUC of 0.923.

### 3.4 Interpretable machine learning and individual risk prediction

**Figure 3:**
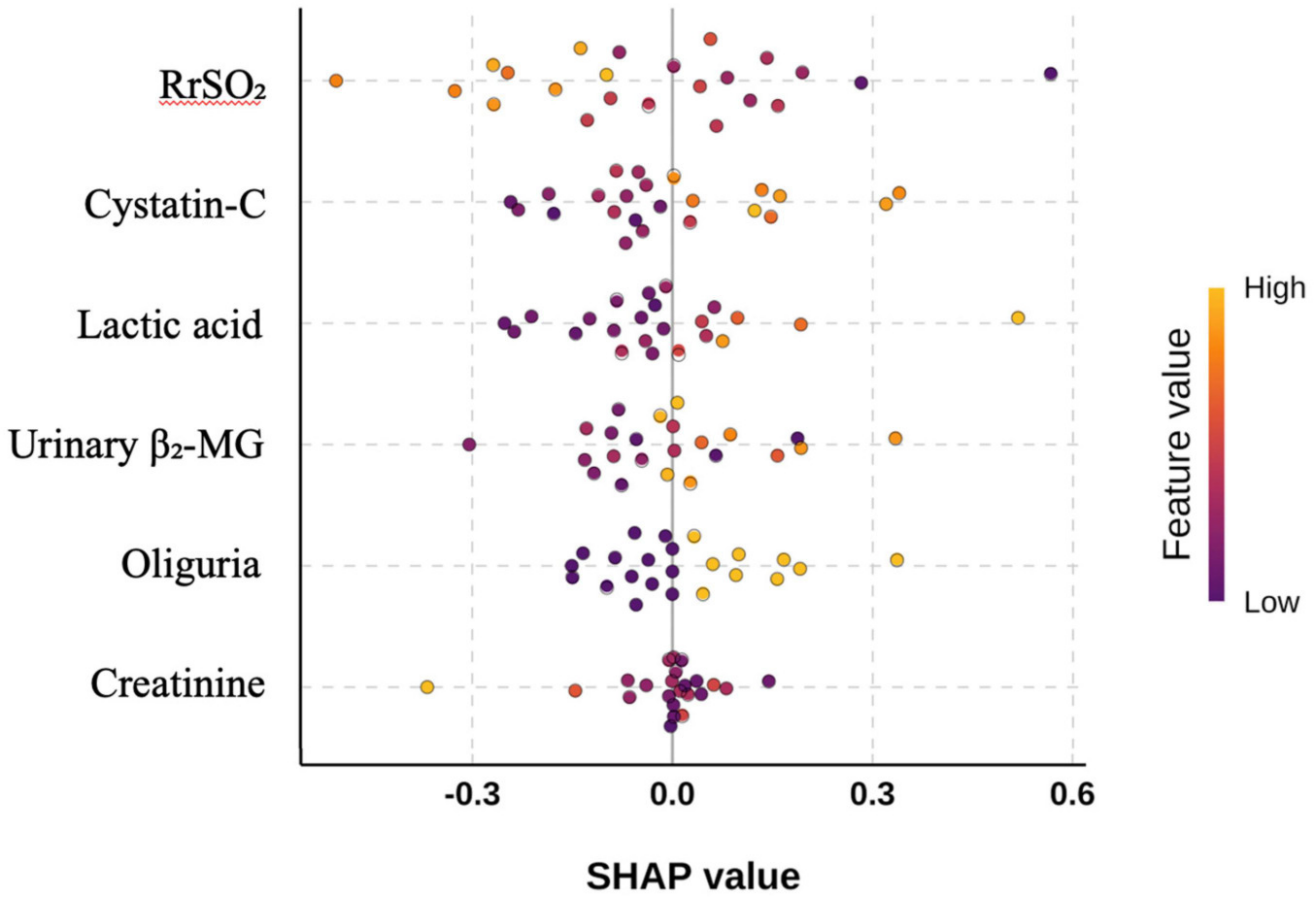
The beeswarm plot obtained by analyzing the model using an interpretable machine learning method.

**Figure 4:**
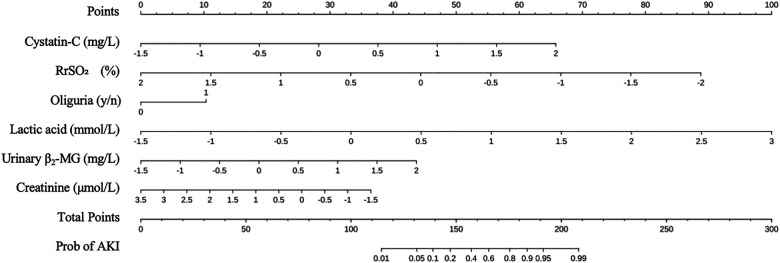
Nomogram of AKI prediction model.

Figure 3 show that Cystatin-C, lactic acid, urinary β_2_-MG, and oliguria exhibit relatively high SHAP values under high feature values, making positive contributions to AKI risk prediction. In contrast, RrSO_2_ and creatinine show an opposite contribution pattern in the model: higher feature values of these two indicators significantly reduce the probability of AKI. The nomogram in Figure 4 further translates the aforementioned variables into clinically usable risk scores, providing an intuitive and quantifiable decision-making basis for individualized AKI early warning and intervention.

## 4 Discussion

Asphyxiated neonates can release large amounts of catecholamines that stimulate vasoconstriction, leading to a significant decrease in renal blood flow and causing ischemic-hypoxic damage, which can further trigger AKI. Studies have reported that neonatal asphyxia is a risk factor for AKI (12), and the combination of neonatal asphyxia and AKI significantly increases mortality and disability rates in affected infants. Foreign literature reports that the incidence of AKI in asphyxiated neonates can be as high as 54.00%–61.86% (1). Our study shows that the incidence of AKI in asphyxiated neonates is 43.03%, indicating a high risk of AKI in this population. Therefore, accurately identifying early AKI is of great clinical significance for developing targeted treatment plans, controlling disease progression, and reducing neonatal mortality.

Cystatin-C is a cysteine protease inhibitor containing 122 amino acids, produced by nucleated cells without tissue specificity. The kidneys are the sole excretory organ for Cystatin-C in the body, which can freely filter through the glomeruli and is rapidly degraded after reabsorption in the proximal tubules without re-entering the bloodstream (13). Serum Cystatin-C is a more accurate estimate of neonatal GFR. With a small molecular weight, Cystatin-C concentration is determined by glomerular filtration function and is not affected by external factors such as gender, age, or diet. It is an early endogenous indicator of early glomerular filtration function. Numerous studies have shown that Cystatin-C is not only a biomarker for the diagnosis of AKI but also plays an important role in assessing disease severity and monitoring prognosis (14). Our study found that serum Cystatin-C levels were significantly higher in the AKI group than in the non-AKI group, and the RFECV results showed that Cystatin-C is an important variable for predicting AKI, with an importance score of 19.95. This suggests that in asphyxiated neonates, impaired glomerular filtration function leads to elevated Cystatin-C levels, which can serve as an important basis for early AKI prediction.

Near-infrared spectroscopy (NIRS) was first clinically applied in the 1980s for monitoring local tissue oxygenation in neonates. By measuring the mixed arteriovenous weighted average in the local tissue microcirculation, it reflects the balance of tissue organ oxygen supply and demand as well as hemodynamic changes (15). Current extensive research indicates that NIRS can detect renal perfusion insufficiency early and is a sensitive indicator for monitoring renal function, even superior to traditional biochemical markers. RrSO_2_, as an important indicator of renal tissue oxygen metabolism, showed in our study that RrSO_2_ levels were lower in the AKI group than in the non-AKI group (*P* < 0.05), indicating a close relationship between RrSO_2_ and AKI. Asphyxia leads to renal tissue hypoxia, which in turn causes a decrease in RrSO_2_. The more severe the renal tissue hypoxia, the higher the risk of AKI. The RFECV algorithm also showed that RrSO_2_ is an important predictive variable for AKI, which is consistent with the pathological and physiological mechanisms of renal susceptibility to damage under hypoxic conditions.

Our study found that oliguria is an important predictive variable for AKI and is significantly associated with the occurrence and development of AKI. This result is highly consistent with clinical observations and pathological and physiological mechanisms. Oliguria is one of the core clinical manifestations of AKI, essentially reflecting the dual pathological processes of a sharp decline in GFR and impaired renal tubular function (16). The vicious cycle between oliguria and AKI has been widely verified. Oliguria can lead to fluid retention, electrolyte disturbances, and accumulation of uremic toxins, further exacerbating renal ischemia-reperfusion injury and oxidative stress reactions, forming a positive feedback loop of oliguria-renal damage-worsening oliguria (17). Our study quantified the predictive value of oliguria through the RFECV model, providing an objective basis for early identification of high-risk patients with AKI in clinical practice.

Lactate is a product of anaerobic glycolysis in tissues under hypoxia, and its elevated levels reflect the state of tissue hypoxia. Previous studies have shown that arterial blood lactate is associated with the severity of neonatal asphyxia. After neonatal asphyxia, lactate accumulates in the body, leading to metabolic acidosis. The more severe the asphyxia, the higher the arterial blood lactate levels (18), which is consistent with the results of our study. In our study, serum lactate levels were significantly higher in the AKI group than in the non-AKI group (*P* < 0.05), and RFECV also identified lactate as an important predictive variable for AKI, indicating that lactate levels can serve as a key early warning indicator for assessing the degree of renal tissue hypoxia and the risk of AKI.

Our study results showed that urinary β_2_-MG levels were higher in the AKI group than in the non-AKI group (*P* < 0.05), suggesting that urinary β_2_-MG may be involved in the occurrence and development process of AKI in asphyxiated neonates. β_2_-MG is associated with the risk of end-stage renal disease and death. It is a low-molecular-weight protein that is freely filtered through the glomerular membrane and metabolized in the renal tubules (19). However, most of the filtered protein is reabsorbed in the proximal tubules, resulting in negligible concentrations of β_2_-MG in urine (20). Renal injury secondary to asphyxia mainly affects tubular cells, leading to tubular dilation, vacuolization, increased eosinophilic cells in the tubules, and brush border detachment and fragmentation. Elevated urinary β_2_-MG levels in asphyxiated neonates may indicate proximal tubular dysfunction (21). Urinary β_2_-MG concentration is a sensitive indicator for detecting renal tubular function in asphyxiated neonates, and urinary β_2_-MG as an early marker of AKI in perinatal asphyxiated neonates can assist clinicians in accurately assessing AKI in asphyxiated neonates. Urinary β_2_-MG levels are higher in AKI patients, and significantly elevated in severely asphyxiated patients.

Traditionally, SCr has been used for the diagnosis and staging of AKI in children (22, 23). Currently, the diagnosis of neonatal renal injury mainly relies on clinical symptoms such as oliguria and hematuria, and elevated levels of serum creatinine and blood urea nitrogen. However, early clinical symptoms of neonatal renal injury are atypical, and serum creatinine and blood urea nitrogen can only reflect overall renal function damage, not early renal injury. When changes in serum creatinine and blood urea nitrogen occur, renal function damage is often already at a certain level. The limitations of using SCr as a renal function indicator have been widely discussed. Neonatal SCr can reflect maternal levels and may take a week or longer to return to baseline according to gestational age. Creatinine levels vary with children's muscle mass. Importantly, SCr is a marker of function, not damage (24). Neonatal serum SCr changes are lagging, and when its levels abnormally rise, AKI has significantly worsened, leading to missed opportunities for optimal treatment and delayed diagnosis (25). Our study showed no statistical difference in SCr levels between the asphyxiated AKI and non-AKI groups (*P* > 0.05), consistent with the aforementioned studies. However, SCr can be used in combination with other variables to predict AKI in neonates.

Compared with previous studies (26), our study is the first to integrate NIRS renal oxygen monitoring with multiple biomarkers, using the RFECV algorithm to screen the optimal set of predictive variables and assess variable importance, and finally predict AKI in children using a Bayesian classifier. The model we established achieved good predictive performance, with an AUC of 0.987 in the original test set and an AUC of 0.923 in the test set. In the future, clinicians can assess the risk of early AKI occurrence by detecting relevant physiological and biochemical indicators in asphyxiated neonates and combining them with optimal cutoff values, guiding doctors to develop targeted treatment plans, preventing neonatal renal function deterioration, reducing AKI occurrence, and ultimately improving prognosis.

However, our study also has certain limitations. First, the sample size is relatively small, which may introduce some sampling bias. Future studies need to further expand the sample size and conduct multicenter studies to verify the accuracy and generalizability of the model. Second, our study only detected relevant indicators within 6 h after birth, without dynamic monitoring of these indicators, which may not fully reflect the occurrence and development process of AKI. Future studies can consider dynamic monitoring of relevant indicators and combining more potential risk factors to further improve the risk prediction model.

## 5 Conclusion

Our study combined SMOTE resampling and RFECV, using five—fold cross—validation to successfully construct an early AKI risk prediction model for asphyxiated neonates, which comprehensively considered indicators such as Cystatin-C, RrSO_2_, Oliguria, Lactic acid, urinary β_2_-MG, and Creatinine. The established model had good predictive performance in the test set. The results of our study can be used to clinically assess the risk of early AKI in asphyxiated neonates, providing a scientific basis for early intervention. Further research is still needed in the future to refine and validate this model, so that it can be better applied in clinical practice and improve the prognosis of asphyxiated neonates.

## Data Availability

The original contributions presented in the study are included in the article/Supplementary Material, further inquiries can be directed to the corresponding author.

## References

[1] Gedefaw GD Abuhay AG Endeshaw YS Birhan MA Ayenew ME Genet GB Incidence and predictors of acute kidney injury among asphyxiated neonates in comprehensive specialized hospitals, northwest Ethiopia, 2023. Sci Rep. (2024) 14(1):16480. 10.1038/s41598-024-66242-3 39013957 PMC11252324

[2] Wood S Crawford S Hicks M Mohammad K. Hospital-related, maternal, and fetal risk factors for neonatal asphyxia and moderate or severe hypoxic-ischemic encephalopathy: a retrospective cohort study. J Matern Fetal Neonatal Med. (2021) 34(9):1448–53. 10.1080/14767058.2019.1638901 31331211

[3] Aziz K Lee HC Escobedo MB Hoover AV Kamath-Rayne BD Kapadia VS Part 5: neonatal resuscitation: 2020 American Heart Association guidelines for cardiopulmonary resuscitation and emergency cardiovascular care. Circulation. (2020) 142(16_suppl_2):S524–50. 10.1161/CIR.0000000000000902 33081528

[4] Askenazi D Abitbol C Boohaker L Griffin R Raina R Dower J Optimizing the AKI definition during first postnatal week using assessment of worldwide acute kidney injury epidemiology in neonates (AWAKEN) cohort. Pediatr Res. (2019) 85(3):329–38. 10.1038/s41390-018-0249-8 30643188 PMC6377843

[5] Mok TYD Tseng MH Lee JC Chou YC Lien R Lai MY A retrospective study on the incidence of acute kidney injury and its early prediction using troponin-I in cooled asphyxiated neonates. Sci Rep. (2020) 10(1):15682. 10.1038/s41598-020-72717-w 32973292 PMC7519155

[6] Bozkurt O Yucesoy E. Acute kidney injury in neonates with perinatal asphyxia receiving therapeutic hypothermia. Am J Perinatol. (2021) 38(9):922–9. 10.1055/s-0039-1701024 31986537

[7] Lazarovits G Ofek Shlomai N Kheir R Bdolah Abram T Eventov Friedman S Volovelsky O. Acute kidney injury in very low birth weight infants: a major morbidity and mortality risk factor. Children. (2023) 10(2):242. 10.3390/children10020242 36832371 PMC9955621

[8] Dzakpasu S Joseph KS Huang L Allen A Sauve R Young D. Decreasing diagnoses of birth asphyxia in Canada: fact or artifact. Pediatrics. (2009) 123(4):e668–72. 10.1542/peds.2008-2579 19336357

[9] Zarbock A John S Jorres A Kindgen-Milles D, Kidney Disease: Improving Global Outcome. New KDIGO guidelines on acute kidney injury. Practical recommendations. Anaesthesist. (2014) 63(7):578–88. 10.1007/s00101-014-2344-5; Neue KDIGO-Leitlinien zur akuten Nierenschadigung. Praktische Handlungsempfehlungen. 24981152

[10] Chawla NV Bowyer KW Hall LO Kegelmeyer WP. SMOTE: synthetic minority over-sampling technique. J Artif Intell Res. (2002) 16:321–57. 10.1613/jair.953

[11] Misra P Yadav AS. Improving the classification accuracy using recursive feature elimination with cross-validation. Int J Emerg Technol. (2020) 11(3):659–65.

[12] Ramya K Mukhopadhyay K Kumar J. Predictive factors and risk scoring system for acute kidney injury (aki) in sick neonates-a prospective cohort study. Eur J Pediatr. (2024) 183(12):5419–24. 10.1007/s00431-024-05816-9 39407040

[13] Shlipak MG Inker LA Coresh J. Serum cystatin C for estimation of GFR. J Am Med Assoc. (2022) 328(9):883–4. 10.1001/jama.2022.12407 PMC12790772 35939309

[14] Ding L Liu Z Wang J. Role of cystatin C in urogenital malignancy. Front Endocrinol. (2022) 13:1082871. 10.3389/fendo.2022.1082871 PMC9794607 36589819

[15] Saito J Takekawa D Kawaguchi J Suganuma T Konno M Noguchi S Preoperative cerebral and renal oxygen saturation and clinical outcomes in pediatric patients with congenital heart disease. J Clin Monit Comput. (2019) 33(6):1015–22. 10.1007/s10877-019-00260-9 30666542

[16] Meyrier A Niaudet P. Acute kidney injury complicating nephrotic syndrome of minimal change disease. Kidney Int. (2018) 94(5):861–9. 10.1016/j.kint.2018.04.024 29980292

[17] Hoste EA Bagshaw SM Bellomo R Cely CM Colman R Cruz DN Epidemiology of acute kidney injury in critically ill patients: the multinational AKI-EPI study. Intensive Care Med. (2015) 41(8):1411–23. 10.1007/s00134-015-3934-7 26162677

[18] Olofsson P. Umbilical cord pH, blood gases, and lactate at birth: normal values, interpretation, and clinical utility. Am J Obstet Gynecol. (2023) 228(5S):S1222–40. 10.1016/j.ajog.2022.07.001 37164495

[19] Zaleska-Kociecka M Skrobisz A Wojtkowska I Grabowski M Dąbrowski M Kuśmierski K Serum beta-2 microglobulin levels for predicting acute kidney injury complicating aortic valve replacement. Interact Cardiovasc Thorac Surg. (2017) 25(4):533–40. 10.1093/icvts/ivx198 28962501

[20] Kari JA Shalaby MA Sofyani K Sanad AS Ossra AF Halabi RS Urinary neutrophil gelatinase-associated lipocalin (NGAL) and serum cystatin C measurements for early diagnosis of acute kidney injury in children admitted to PICU. World J Pediatr. (2018) 14(2):134–42. 10.1007/s12519-017-0110-x 29464581

[21] Sarafidis K Tsepkentzi E Agakidou E Diamanti E Taparkou A Soubasi V Serum and urine acute kidney injury biomarkers in asphyxiated neonates. Pediatr Nephrol. (2012) 27(9):1575–82. 10.1007/s00467-012-2162-4 22532328

[22] Soler YA Nieves-Plaza M Prieto M Garcia-De Jesus R Suarez-Rivera M. Pediatric risk, injury, failure, loss, end-stage renal disease score identifies acute kidney injury and predicts mortality in critically ill children: a prospective study. Pediatr Crit Care Med. (2013) 14(4):e189–95. 10.1097/PCC.0b013e3182745675 23439463 PMC4238883

[23] Khwaja A. KDIGO clinical practice guidelines for acute kidney injury. Nephron Clin Pract. (2012) 120(4):c179–84. 10.1159/000339789 22890468

[24] Cirillo L De Chiara L Innocenti S Errichiello C Romagnani P Becherucci F. Chronic kidney disease in children: an update. Clin Kidney J. (2023) 16(10):1600–11. 10.1093/ckj/sfad097 37779846 PMC10539214

[25] Jetton JG. Neonatal acute kidney injury: the signal is clear. It is time to move the field forward. Pediatr Crit Care Med. (2016) 17(4):376–8. 10.1097/PCC.0000000000000686 27043907

[26] Zhang Y Zhang B Wang D Shi W Zheng A. Evaluation of novel biomarkers for early diagnosis of acute kidney injury in asphyxiated full-term newborns: a case-control study. Med Princ Pract. (2020) 29(3):285–91. 10.1159/000503555 31536999 PMC7315142

